# Analysis of the factors influencing the effectiveness of local government’s purchase of older adults care services – a grounded theory study based on typical cases

**DOI:** 10.3389/fpubh.2023.1202472

**Published:** 2023-08-10

**Authors:** Sujun Liu

**Affiliations:** School of Political Science and Public Administration, China University of Political Science and Law, Beijing, China

**Keywords:** older adults care services, aging, government, influencing factors, grounded theory

## Abstract

**Background:**

Population aging is a basic national condition in China at present and for a long time to come, forcing the country to accelerate the pace of building its public older adults care system. The government’s purchase of older adults care services has become an effective way to make up for the lack of the family’s older adults care function, to which the Chinese government attaches particular importance. The article selects 11 typical cases from the excellent case base released by the Chinese Ministry of Civil Affairs officials in 2022 to study the influencing factors of the effect of local government purchase of older adults care service supply.

**Methods:**

NVivo data analysis tools have significant advantages in retrieving, analyzing and coding data more efficiently and accurately, which helps to construct theoretical propositions and formulate hypotheses to be tested in qualitative research. The study intends to adopt the grounded theory approach to analyze the text with the help of NVivo12 software, to condense the practice mechanism of local governments’ purchasing of older adults care services and to construct a relational model.

**Results:**

Taking “the supply effect of local government purchasing older adults services” as the main logic line, the article summarizes the four main influencing factors of the supply effect of government purchasing older adults services: the real demand of the society, the government’s power and responsibility system, the government’s governance ability, and the society’s acceptance ability.

**Conclusion:**

The sense of gain, happiness and security of the older adults group is the starting point and landing point of the older adults service policy formulation and implementation. Policy guidance and decision-making have an important impact on the quality of the supply of older adults care services and the development of the older adults care services industry. Clarifying the direction of policy guidance, reflecting the comprehensive efficiency of government governance and utilizing the professional advantages of social forces, is the key to improving the effectiveness of the government’s purchase of older adults care services.

## 1. Introduction

At present, China’s local governments are facing many difficulties in purchasing older adults care services, including imbalance between supply and demand, stagnant mechanism, escalating costs and risk (1–8). Against the backdrop of the structural contradiction between the increasingly serious problem of an aging population and the weakening function of family care, the purchase of older adults care services by the government has received increasing attention from academics (9–18). The first attempt by a local government to purchase older adults care services in China began in 2003, when Shanghai pioneered a policy of government purchase of older adults care services at home, and in 2013 the General Office of the State Council issued the “Guidance on the Purchase of Services by the Government from Social Forces,” proposing that “in basic public services such as education, employment, older adults care and disability assistance, social forces should be leveraged to increase in 2014, the Ministry of Finance and other departments issued the Notice on the Government’s Purchase of Senior Care Services, which requires “taking the basic senior care service needs of the older adults as the guide and linking the government’s purchase of services to meeting the basic senior care service needs of the older adults.” In 2014, the Ministry of Finance and other departments issued a circular on the government’s purchase of senior care services, requiring the government to “combine the purchase of services with meeting the basic senior care service needs of the older adults, giving priority to the needs of widows, orphans, the disabled and the older adults.” Under the guidance of the national top-level design, local governments across the country have started to intensively introduce local regulations and policies on government purchase of older adults care services, and the local governments’ purchase of older adults care services has continued to increase in strength and expand in scope (19).

At present, 31 provinces (municipalities) in mainland China have issued guiding documents on government purchase of older adults care services, exploring and piloting the implementation of government purchase of community-based older adults care services from social organizations to provide older adults care services, and pilot work on older adults care services at home has been carried out in Shanghai, Beijing, Jiangsu, Zhejiang, Nanjing, Qingdao, Dalian, Harbin and Guangzhou in China, and the Shanghai model, the Nanjing Gulou model, Anhui Province Hefei model, Ningbo Haishu model and many other representative operational models. However, with the increase in the number and scope of government purchase of older adults care services, a series of problems have been exposed, with some areas even facing the problem of “hollowing out” of the purchased services. Some scholars have even called it an “unsatisfactory gift” from the public (20). To avoid the spread of “hollowing out,” the phenomenon of “reverse contracting out” has emerged in some reform areas (21).

From the existing literature, there is a growing body of research on government purchase of older adults care services in China, but these studies have mainly focused on the introduction of local practical experiences (22, 23), typical models of service provision (24–27), potential risks (28) and the exploration of the boundary between government and society (29), with little focus on the core issue of the factors influencing the effectiveness of government purchase of older adults care services provision. Therefore, the research question in this paper is: why does the purchase of older adults care services by local governments show differential effects, despite the same high level of central policy, the constraints of limited resources of local governments, and the same weak economic preference attributes? What are the factors that influence the effectiveness of local government purchase of older adults care service provision?

Generally speaking, only through a large number of empirical surveys and multi-case statistics can we gain insight into the factors influencing government purchase of older adults care services, but this work has not yet been adequately carried out in China. In this regard, this paper selects from the list of “excellent cases of home and community-based older adults service reform pilot work” published by the General Office of the Ministry of Civil Affairs and the General Office of the Ministry of Finance in 2022, Fengtai District, Beijing; Nantong City, Jiangsu Province; Anshan City, Liaoning Province; Changning District, Shanghai; Jiangning District, Nanjing City, Jiangsu Province; Xihu District, Hangzhou City; Anqing City, Anhui Province; Xiashan District, Weifang City, Shandong Province; and the city of Weifang, Shandong Province, the 11 outstanding cases of government purchase of older adults care services in Weifang Xiashan District, Shandong Province, Weihai City, Shandong Province, and Beilin District, Xi’an City, Shaanxi Province were studied, and textual analysis was conducted with the help of NVivo12 software to answer the above questions using a grounded theory approach, systematically analyse the practical picture of local government purchase of older adults care services in China, and provide a reference for the next practical development.

## 2. Literature review

The field of government purchase of older adults care services has a great academic attraction due to its grand government-society relationship, active inter-organizational behavioral strategies and rich and vivid scenario narratives. Current academic research on government purchase of older adults care services focuses on three levels: macro-level government-society synergy, meso-level government purchase behavior and micro-level “supply and demand bias.” Macro-level research focuses on the synergistic relationship between the government and society in socialized older adults care services from a holistic perspective, focusing on the roles and responsibilities of the government in older adults care services; meso-level research focuses on the government’s decision-making and behaviors in the process of purchasing older adults care services, with the researchers exploring the goals, strategies, risks and implementation methods of the government’s purchasing of care services and analyzing the impact of the government’s purchasing behavior on service supply and service quality; micro-level research focuses on the relationship between government supply and demand for individual older adults care services, analyzing the government’s supply capacity and level of supply and the demand for older adults care services, and examining the deviations and imbalances between supply and demand. Specific discussions are as follows:

### 2.1. Macro level - research on the synergy between government and society

The accelerated development of population aging and the advent of a geriatric society are the most irreversible trends in the world today, and have become social problems shared by all countries in the world. From the point of view of worldwide research, the older adults services in different countries have different characteristics, and the diversity of the social service system for the older adults reflects the dynamic combination of the state, the market, the society and the family, behind which there is a long-term connection between the economy, politics and social culture. However, in terms of the overall development trend worldwide, services for the older adults have gradually evolved from a single-care model of the family to one in which the market, the State and the family all participate. To date, the multi-care model of family, market, state and the non-profit sector or other social and civil society organizations has become mainstream (30). The reform of the marketization of older adults care services has changed the role of local governments from a single provider of older adults care services to both a purchaser and a provider. The market has gradually entered local governments and local governments have entered the market, which means that the line between public and private has become more blurred (31).

Government purchasing of older adults care services is one of the most important ways for governments to socialize the older adults, and its positive social functioning relies on a good synergy between government and social organizations. Synergy usually combines complementarity and embeddedness and is most easily promoted in societies characterized by egalitarian social structures and strong, coherent state bureaucracies (32). Current government policies emphasize collaboration and “embeddedness,” particularly between health and social care services (33). Theoretically, the two should be synergistic relationships that are mutually beneficial (34–36), but in practice, the relationship between the two is not very harmonious (37–39), and there are “alienated cooperation,” “organizational convergence” and “cooperation involution” in the process of interaction between “government and society.” “The root cause of this is the failure of government-society interaction” (40). Due to their own lack of capacity and the government’s imperfect policy support system, social organizations face the dilemma of over-reliance on the government’s unevenness and insufficient resources for sustainable development (41), and the formal care system is severely undersupplied and fragmented (42). Practice shows that behind the market logic of strict contract management and emphasis on performance advocated in the purchase of social services, the logic of the section system plays a more important role (43). Local authorities have created structures based on the separation of purchasers and providers and have made for-profit or not-for-profit services, or both, available in place of public services. The language of social care policy is changing to include terms such as choice, client-orientation, partnerships, contracts and markets (44). The role of government has shifted from that of traditional welfare producer to that of planner, purchaser and supervisor, but in its new role assumption it also faces challenges such as poor planning authority and enforceability, inefficient use of funds and lack of effective and viable monitoring mechanisms (45), Yanhua, looking at the behavior of the government-agency dichotomy, suggests that in the early stages of the development of the home care sector, the government must provide policy incentives while increasing regulation of participating agencies to ensure service quality (46). Ding Shejiao et al. similarly argue that even if a third-party evaluation agency is introduced the government must still not completely retreat (47). China is currently in the process of rapid aging, but at the same time, the “absence” of multiple government, market and social actors has become a structural problem in the government’s purchase of older adults care services.

### 2.2. Meso-level − research on government purchasing behavior

Government policies for the purchase of older adults care services have the integrity, relevance, purpose and contextual adaptation that all complex systems should have (48). Researchers have conducted a number of empirical studies on local government purchase of services, and some recent findings suggest that this form of privatization is less politically controversial and more accepted as a method of service delivery (49). Numerous studies have explored why governments choose to privatise. These reasons can often be grouped according to factors related to quantity, quality and cost. The focus explored provides a valuable reference. For example, some of the frequently cited benefits of contracting are lower costs, greater efficiency, increased flexibility, greater competition leading to improved consumer choice, increased efficiency, improved quality of care, reduced bureaucratic red tape and access to specific expertise (50). Governments have accepted that increasing market competition and, more recently, allowing users to choose services will lead to more personalized services and “improve” the quality of care, as people cannot be forced or incentivized to take care (51). The shift from an integrated system of government finance and provision to a competitive contracting system for the NHS in the United Kingdom is a leading example of an international trend (52). Government policies to purchase social services have been successful in achieving ‘embedded development’ (53), and Fionk Richard et al. offer an explanation in terms of transaction cost theory, arguing that non-profit contracts are more likely to occur when transaction costs are low; when the political system and the structure of the service market result in high transaction costs, the government will produce services for older people internally rather than contract with non-profit providers. Some scholars have argued that contract management capacity is an important determinant of local government services for the older adults (54). Public trust in government is expected to facilitate the effective delivery of public services (55–57). Other scholars have argued that there is a symbiotic evolutionary relationship between local governments and public health service organizations under different reward and punishment mechanisms (58, 59), and that how to reduce the quality risk through ex ante policy design is key (60). There are also studies that suggest that government purchasing power and diversity of regulatory agencies are the primary considerations for government purchasing of older adults services, with the remaining vulnerability influences ranking second and third (61).

There is a clear trend towards seeking to integrate long-term geriatric care with curative and preventive care, especially in community-based settings. Integration is reflected not only horizontally but also vertically, transcending public and private boundaries (62). Social support and social connectedness are critical to the health and well-being of the older population (63). Good social care bridges the gap between the healthcare system and the social support system, and can also promote the use of long-term care services in the community through recreational activities. Notably, social engagement in recreational activities can help promote and improve integrated care services (64–66).

Under the influence of Western social welfare pluralism, the Chinese government’s purchase has become a new pathway for the provision of institutional older adults care services, with the government gradually changing from a “direct provider of services” to an “indirect provider of services.” In China, there are three main models: the public private sector, the private government, and the public subsidized private sector (67). Other scholars argue that, in terms of commission-agent relationship, social work agencies, as the main private organizations that the government purchases community older adults care services from, are subject to both “contractual commissioning” by the civil affairs department and “administrative commissioning” by the street offices, which can easily lead to multiple This can lead to multiple information asymmetries and differences in value orientations among multiple parties (68). Romzek and others have explored the effectiveness of contractual responsibility in social service contracts through a number of cases where government accountability has been weakened by the use of risk transfer, systems that rely on multiple competing providers and the adoption of new information technologies (69). In particular, since the 12th Five-Year Plan (2011–2015), the central government has increased its efforts to create a market for older adults care services (Du, 2013: 59) (70). On the other hand, in China’s institutional environment, public participation is inadequate and the government’s purchase of public services is guided by the traditional sectoral and administrative preferences in public service provision, which can easily lead to a deviation between public services and public demand. Therefore, the effectiveness of government purchase of public services has become the central issue at hand (71). Some Scholars have also examined the reasons for government purchases of public services, suggesting that corporatization, public-private partnerships (PPP) and public-private partnerships (PPP) are the drivers of the externalization options for public service delivery, and that a combination of financial and economic efficiencies are the factors behind externalization. In addition, political ideology influences the choice of PPP (72). While some scholars in the current academic community argue that governments have the potential to increase overall social welfare through social public purchasing, little is known about how indirect purchasing can be utilized to meet social needs (73).

### 2.3. Micro-level − “supply and demand bias” studies

That population aging is a social problem faced by many countries around the world (74–77), and should be given high priority on the agendas of local and national Governments. All sectors should be involved in adapting care systems and programs to the rapid growth of older persons and the relative balance of age groups in the population (78, 79). Severe population aging has led to a rapid increase in demand for urban older adults care services in most countries (80–83). There is a certain degree of deviation between the supply of government-purchased older adults care services and the actual needs of the older adults. The imbalance in service provision and the exploratory design of the insurance system remain to be resolved (84, 85). The level of imbalance in older adults care services began to increase in 2009 and further intensified in 2011. Dongsheng and Yanfang used both matrix scatter plots and regression analysis to show that the imbalance between supply and demand for older adults care services in China is significantly related to the funding of older adults care services, the number of institutions providing older adults care services and the number of professionals in older adults care services (86). The ‘supply–demand bias’ between government supply and older adults demand is mainly reflected in the following: firstly, the government’s purchase of family services for the older adults may be ethically risky and inefficient. The use of policy to establish the relationship between parents and children in the purchase of services is contrary to the natural harmony of kinship and ethical relationships, and an unreasonable policy design that ignores the ethics of kinship will affect the objectivity and fairness of the assessment of older adults care services (87). Secondly, the traditional criteria for classifying older adults in poverty can no longer be adapted to the reality of the older adults, and the main beneficiary group of the older adults service support policy has changed (88, 89). There is a mismatch in expectations and perceptions of integrated care between service providers and older users, users are often less aware of the need for team-based care and the links between their social and medical needs (90–92). Addressing governance and management issues is critical to the development of care for older people (93). Therefore, local governments should, on the basis of adhering to the ethical value of fairness and justice in social welfare distribution, clearly define the general and specific scope of government purchase of older adults care services, reasonably configure the subsidy standards for older adults care services, and formulate long-term phased plans to cover older adults from low-income families in urban and rural areas, so as to ultimately promote the purpose of providing for all members of society (94). Other scholars, drawing on the views of new institutionalism, believe that the root of the institutional dilemma lies in the government’s failure to provide social organizations with suitable institutional norms in the process of transferring older adults care functions, and that the government, as the main body of institutional supply, should take into account the socio-cultural background and the characteristics of older adults care services, and carry out institutional innovation in two aspects, namely optimizing regulatory elements and internalizing cultural-cognitive elements (95). The above scholars have taken the pulse of the problem of “supply–demand discrepancy” between government purchase and the needs of the older adults, and have prescribed precise remedies.

In conclusion, the existing studies have provided useful reference for the academic community to explore the research on coping with aging, and have made an important contribution to the research on the government’s purchase of older adults services. These studies provide valuable insights into the factors affecting the effectiveness of government purchasing of services, and reveal the different roles of the government and social forces in government purchasing of older adults services, the potential risks in the process of cooperation, as well as the problem of “supply and demand bias” between the government and the older adults. The shortcomings are that the established studies focus on the description of the theoretical level of the government’s purchasing of older adults services as a matter of course, lack the description of diversified practices in different regions, and most of them sever the influencing factors from the differentiated effects, lacking a more complete research logic. There are fewer studies on the influencing factors of the effect of the government’s purchase of older adults services supply, and fewer empirical studies on the government’s purchase of older adults services, especially not discussed in the special context of China’s large population size and rapid aging rate. Although some relevant studies have been conducted in China, there are not many focused studies on excellent national-level cases utilizing the grounded theory approach, which happens to be a key research gap that can be filled by public management scholars.

Based on this, this paper will do further research, using the grounded theory research methodology, starting from the practical experience of Chinese regions, and selecting 11 typical representative cases of government purchase of older adults services from the list of excellent cases of home and community-based older adults service reform pilots published by the Ministry of Civil Affairs, to respond to the influencing factors affecting the purchase of older adults services by the local government as well as the inherent cause and effect mechanism on the doctrinal level, and to strive to this study seeks to make up for the lack of attention to the actual situation in existing studies, and to enrich the theoretical value and management practice of the study of government purchase of senior care services. At the same time, this study also helps to re-examine the problems in the governance of multiple supplying subjects in the government’s purchase of older adults services at the practical level, and provides theoretical support and policy suggestions for the realization of benign governance of multiple subjects in the government’s purchase of older adults services, so as to enhance the effectiveness of the governance of the local government’s response to aging.

## 3. Research design

In this paper, 11 excellent cases were selected, and the text content was analysed using NVivo12 software, and the data content was coded at three levels of open coding, main axis coding and selective coding with the help of the rooting theory method, and further theoretical saturation verification was made, and finally an impact relationship model was established.

### 3.1. Data sources

“During the 13th Five-Year Plan period, the Ministry of Civil Affairs and the Ministry of Finance” have selected five batches of pilot projects in 203 areas across the country, and in terms of regional distribution, the five batches of pilot projects cover 26 cities (districts) in 20 provinces, 28 cities (districts) in 17 provinces, 36 cities in 22 provinces, 54 cities in 30 provinces, 59 cities in 30 provinces, and Lhasa in the Tibet Autonomous Region. In March 2022, the General Office of the Ministry of Civil Affairs and the General Office of the Ministry of Finance announced the list of outstanding cases of home and community-based older adults service reform. 51 outstanding cases were selected to bring together the advanced experience of home and community-based older adults service reform across the country. In this paper, we have selected 11 typical representative cases of government purchase of older adults services for study, and the principles of case selection are “authority, typicality, innovation, diversity, and case completeness,” which are as follows: (1) Authority: The Ministry of Civil Affairs (MCA) is an important department of the Chinese government responsible for the supervision and guidance of the reform of the home-based and community-based older adults services. Supervision and guidance of the reform. The list of excellent cases published by the Ministry of Civil Affairs is an authoritative source recognized nationwide, with strong credibility and representativeness. (2) Typicality: After evaluation and screening, these 11 samples selected in this paper represent typical experiences that have achieved remarkable results in the field of home and community-based older adults service reform. The practical feasibility of these cases has been verified, and they can provide feasible older adults service reform solutions for other regions or organizations. (3) Innovative: These 11 samples are innovative, reflecting the different practices and attempts of various regions in the reform of home and community-based older adults services. By studying these prominent cases, it is possible to understand the experiences and practices of different regions, and extract the success factors and lessons learned from them, so as to provide lessons and references for other regions or organizations. (4) Diversity: The cases selected in this paper cover different regions and different types of older adults service reform cases. Choosing to study 11 prominent cases can ensure that a comprehensive understanding of different regions and types of cases is obtained during the research process, thus broadening the horizon and improving the breadth and depth of the study. (5) Case completeness: a quality case should contain sufficient information and data so that readers can gain an in-depth understanding of the case’s background, objectives, implementation process, outcomes and effects. The relevant information and data of the cases selected for this study are comprehensive, detailed and reliable, and therefore feasible for in-depth research.

As the “9,073” model is the main mode of older adults care in China, i.e., 90% of older adults age at home, 3% in older adults care institutions and 7% in the community, i.e., 97% of older adults choose to age at home or in the community. This means that 97% of the older adults choose to age at home or in the community, therefore, the sample of “excellent cases of home and community older adults service reform” is more representative. The 11 cases selected are shown below:

1. Fengtai District, Beijing: Focusing on the immediate needs of older adults with disabilities and dementia, developing “respite services.”
2. Nantong, Jiangsu Province: Innovative “chain-type older adults care” service model.
3. Wuhan City, Hubei Province: interoperability between reality and reality, mutual integration of home and hospital, exploring a new model of “Internet + home care.”
4. Anshan City, Liaoning Province: Seizing new opportunities to build a new pattern for the development of rural home and community-based older adults care services.
5. Changning District, Shanghai: Focusing on the “Five Firsts” to Improve the Graded Care System for the older adults with Cognitive Impairment.
6. Jiangning District, Nanjing, Jiangsu Province: Coordinated development of business and industry to build a home care service system for the older adults.
7. Xihu District, Hangzhou City, China: Exploring the establishment of “one bed for the older adults” service mechanism through innovation-driven digital intelligence empowerment.
8. Anqing City, Anhui Province: Adhering to the precision and standardization of performance and innovating the institutional mechanism of government purchase services.
9. Xiashan District, Weifang City, Shandong Province: Cracking the problem of old-age care for the disabled in rural areas with “four” construction.
10. Weihai City, Shandong Province: Spreading “three networks” to build a wisdom home care project.
11. Beilin District, Xi’an City, Shaanxi Province: Implementing the “embedded” bed-based older adults care model to solve the problem of older adults care services in the main city.

The study started through the Chinese government website,^1^ local government official websites, China Knowledge Network, news media (including online versions of media), and then snowballed into the relevant websites involved in the case, comprehensively collecting relevant government policy documents, officials’ speeches, research literature, work summaries or reports of relevant departments, mass media reports, etc. The case materials were then compiled into a standardized form that could be used for further academic research. Literature search, word frequency analysis and text analysis were used to extract features from the policy texts and analyse the relationships between the elements.

### 3.2. Research methodology

Grounded theory is a commonly used and influential qualitative research method. Compared with other qualitative research, grounded theory has a complete and relatively standardized set of operational procedures, with special emphasis on summarizing the empirical generalization from the original data and then rising to the theory, which improves the scientific nature of qualitative research and also has strong practicality.

Grounded theory research methodology has a wide range of applications in the social sciences, for example, in the field of education research, Veladat et al. explored the issue of talent management in elementary school from the perspective of education experts, who were asked to respond to an open-ended researcher-designed questionnaire for data collection, and based on grounded theory, conceptualized four main categories from the findings of the questionnaire (96). Also for example in the field of behavioral science, Mahdi Malakoutikhah was conducted in 35 industries using a semistructured interview based on grounded theory, from the interview data organizational factors were the most categorized, and it is estimated that this factor has a more important role in the UBs (97). As another example, in the field of economics, Cheng Guisun et al. based on the social responsibility reports released by 12 local Internet platform enterprises such as Alibaba, DDT, Meituan, etc., applied the grounded theory analysis method and used Nvivo 12.0 software to open coding, spindle coding and selective coding of the raw materials in order to summarize and extract China’s Internet platform corporate social responsibility. In conclusion (98). Grounded theory takes “constructive theory” as its primary meaning: that is to say, through the systematic analysis of qualitative data, it can make a general explanation of social phenomena or problems, explore the correlation between things, and predict the laws and trends, which can make up for the shortcomings of qualitative research in the field of theoretical construction.

NVivo software is one of the qualitative analysis software widely recognized and used by academics (99), and its powerful coding, querying, and categorization functions can help researchers conveniently analyze large amounts of interviews, cases, policies, and other texts, and is commonly used to help social science researchers solve problems that are difficult to solve quantitatively or qualitatively alone. Researchers can use NVivo to annotate, highlight, tag, and code text to identify important themes, patterns, and relationships, which can help uncover hidden information in the data and help researchers better process and analyze their research data and derive valuable insights and findings from it. In short, the NVivo data analysis tool has significant advantages and has the ability to code data beyond manual limitations (100), allowing for more efficient and accurate retrieval, analysis and coding of data (101), helping to construct theoretical propositions and formulate hypotheses to be tested in qualitative research (102). This study adopts the grounded theory approach to carry out research, coding the textual information with the help of NVivo12 software, and abstracting, condensing and categorizing certain important information with the help of the background knowledge of related interdisciplinary disciplines such as public administration and social security. With the help of the grounded theory method, the content of the information was coded at three levels: open coding, spindle coding and selective coding, and the textual information was dissected in depth, so as to concretize the deeper tacit knowledge, in order to better analyze the attributional effects of the government’s purchasing of older adults care services. The research process is divided into three steps:

#### 3.2.1. Open coding

The first step in this study was to conduct open coding of all the information. The requirement of open coding is that the researcher must try to discard personal bias or specific research bias and code and analyse the primary sources in an open and free manner so as to refine new concepts and develop new categories (103). Divided the 260,000-word primary source into 25 document samples and extracted and identified 455 initial utterances and their corresponding initial concepts, of which four-fifths (i.e., 20) were randomly selected for coding and model construction, and the remaining five were used for theoretical saturation tests. The following table excerpts 1–2 original utterances and their corresponding initial concepts (Table 1).

**Table 1:** Categalization of open coding.

Primary category	Original statement (initial concept)
A1 The base of the older adults is large	In November 2020, it reached 15.8 percent, higher than the average level of 13.3 percent in Beijing, and the total number reached 320,000.
A2 The older adults have a high demand for their services	In Jiangning, there is one older adults person in every four people, 98 percent of whom choose home care, and 40 percent of whom live in rural communities in the hilly areas of the area. When the older adults encounter difficult to eat, medical treatment, care, who should find who, how to pay, how to deal with disputes.	……	……
A23 strengthen personnel team building	To form a home care service team combining older adults care personnel, social workers, volunteers, family and social members.
A24 improve the service level of personnel	Strengthen the capacity building of management personnel, medical and health care professionals, and nursing staff, and carry out vocational and technical training and skill evaluation related to health for the older adults.

#### 3.2.2. Main axis coding

Open coding breaks up, splits and interprets the information to obtain different concepts that may be internally related. Main axis coding means discovering the similarities and semantic links between the initial concepts on the basis of open coding, discovering the potential logic between the categories, and constructing the class relationship between the initial concepts by reorganizing and exploring them. In this study, the 24 free nodes obtained from NVivo12 open coding were categorized according to the interrelationship and logical order of the concept levels, and a total of 11 categories were identified, which are summarized in Table 2.

**Table 2:** Main category formed by the spindle encoding.

Fundamental category	Deputy category	Initial category
Social reality needs	B1 Social reality needs	A1 The base of the older adults is large A2 The older adults have a high demand for their services
	B2 Difficulties in life are prominent	A3 The problem of disability and dementia care is prominent A4 The proportion of empty nest and “only generation” older adults has increased
Government power and responsibility system	B3 organization and management system	A5 Party leadership A6 Party leadership
	B4 Division of government responsibility	A7 Service objectives A8 Standardize the service system A9 Construction of a grassroots service mechanism
Government governance capacity	B5 Problem response ability	A10 Development opportunity identification A11 Innovation-driven consciousness A12 Strengthen the concept of service
	B6 System supply capacity	A13 System building A14 Policy subsidy support
	B7 Ability to cooperate in governance	A15 Guiding the participation of social forces A16 Standardize industry operation
	B8 Digital building capacity	A17 Smart service A18 Intelligent platform
	B9 Supervision and management ability	A19 Supervision and evaluation A20 performance appraisal
Social undertaking capacity	B10 Socialization and market-oriented operation	A21 Integrate community pension resources A22 Cultivate older adults care industry clusters
	B11 Professional talent training	A23 Strengthen personnel team building A24 Improve the service level of personnel

#### 3.2.3. Selective coding

Selective coding generally involves five steps: specifying the story line of the information, describing the primary and secondary categories and concepts, testing the initial hypotheses and filling in the conceptual categories that need to be added, selecting the core categories, and establishing systematic links between the core categories and other categories (104). Selective coding uses a large amount of data to filter the codes and extract the core categories, which ‘emerge naturally’ from the open-ended coding and have two main characteristics: the importance of association and frequent recurrence (105). Following the selective coding approach of rooting theory, the core category of “the effectiveness of local government purchase of older adults care services supply” was identified, and the story line around the core category can be broadly summarized as “social reality demand” The four main categories of “governmental authority and responsibility system,” “governmental governance capacity” and “social acceptance capacity” have a significant impact on the effectiveness of the government’s purchase of older adults care services. Among them, the real demand of society is the external driver of the supply of senior care services purchased by the government, “government authority and responsibility system” is the fundamental guarantee factor, “government governance ability” is the direct determinant, “social acceptance ability” the typical relationship structure (story line) of the main categories is shown in Table 3.

**Table 3:** Typical relational structure of the main category.

Typical relationship structure	The connotation of the relationship structure
Social realistic demand – external drive – supply effect	Social realistic demand is the external driving force that affects the supply effect of government purchase of older adults care services.
Government power and responsibility system – fundamental guarantee – supply effect	The government power and responsibility system provides a fundamental guarantee for the supply effect of the government’s purchase of older adults care services.
Government governance ability – direct decision – supply effect	The governance ability of the government directly affects the supply effect of the government’s purchase of pension services, which is the most important part of the four factors.
Social undertaking capacity – necessary support – supply effect	The social undertaking capacity is the necessary support for the supply effect of the government’s purchase of older adults care services. The cooperation and coordination with the government have a significant interaction effect and a positive synergistic effect.

### 3.3. Theoretical saturation validation

This study used an additional four-fifths of the document sample for saturation validation. The results showed that the initial concepts and categories were saturated, and no new categories or class relationships were found. This indicates that the four main categories (social reality needs, governmental power and responsibility system, governmental governance capacity, and social acceptance capacity) obtained from the open coding, main axis coding and selective coding of the sample data based on the grounded theorycoding method have good validity and theoretical saturation, and the analysis results obtained on this basis are complete and rich. These main categories and their counterparts, and the typical relationships thus established, will also serve as a direct basis for the analysis of the influencing factors below.

**Figure 1:**
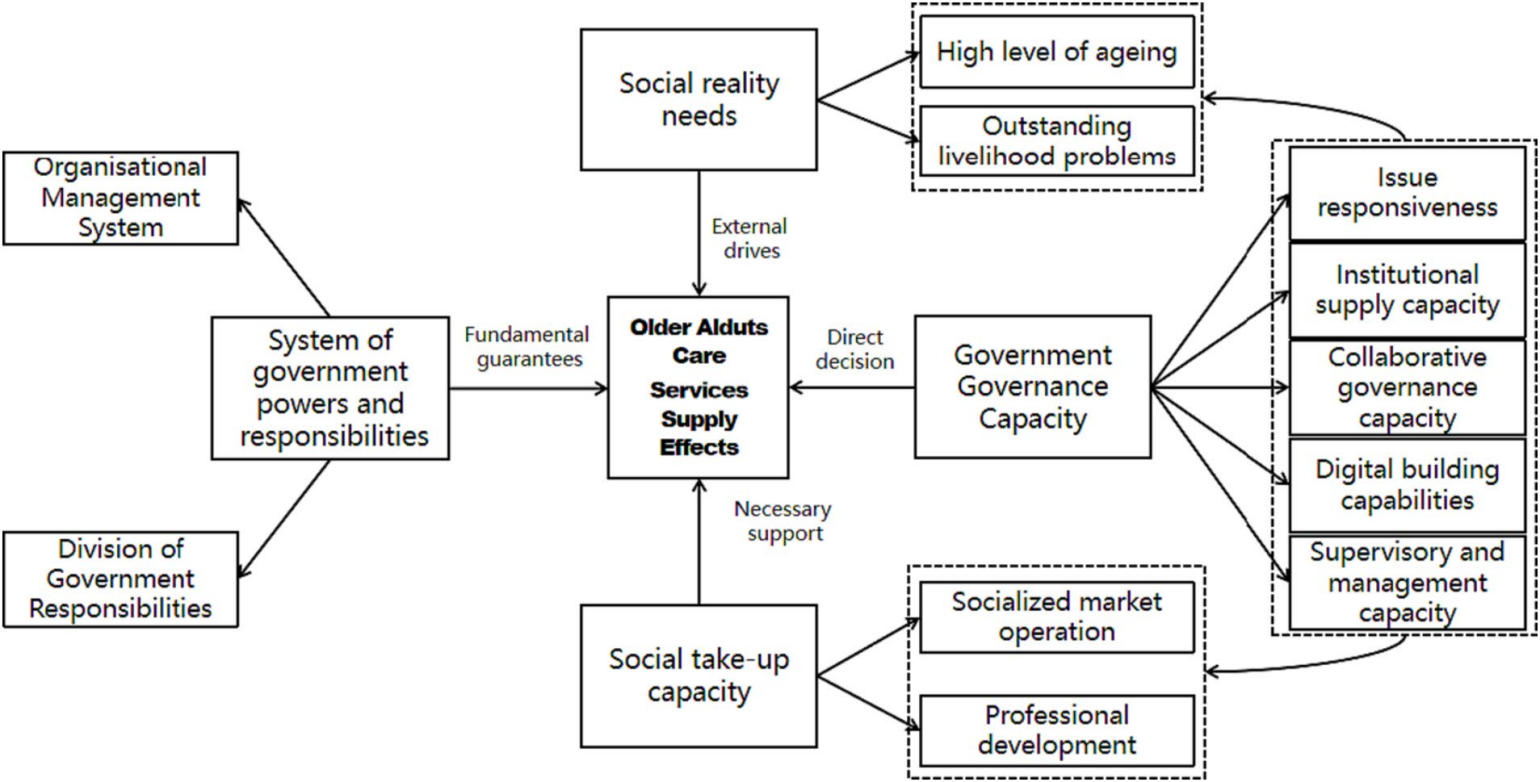
Impact model diagram of the supply effect of government purchase of older adults care services.

This study identifies the core category of “Effectiveness of local government purchase of older adults care services supply,” and the four main categories of “story line” around the core category, i.e., social reality demand, government authority and responsibility system, government governance ability and social acceptance ability, have significant influence on the effectiveness of local government purchase of older adults care services supply. The four main categories of “story line” around the core category, i.e., the real demand of the society, the government’s power and responsibility system, the government’s governance capacity and the social acceptance capacity, have significant influence on the effect of local government purchasing older adults care services supply, and further establish its influence relationship model as shown in Figure 1.

As shown in Figure 1, firstly, the supply effect of government purchased older adults services is driven by social reality demand: due to the serious aging phenomenon and many older adults’s difficult life problems, resulting in the emergence of a large number of socialized older adults service demand, so the social reality demand factor is the external driving factor of the government purchased older adults services. Secondly, the government’s power and responsibility system ensures the supply effect of the government’s purchase of older adults services: the strict organizational management system and the clear division of government responsibilities have built a standardized government service system, which provides a fundamental guarantee for the government’s purchase of older adults services. Once again, the government’s governance capacity is a direct determinant of the supply effect of government-purchased senior care services, which is composed of problem-responsive capacity, system-supplying capacity and cooperative governance capacity, digital construction capacity and supervisory and management capacity, with problem-responsive capacity directly oriented to the needs of the social reality and supervisory and management capacity oriented to the undertaking of the social organizations. Finally, the social undertaking capacity is the necessary support for the effect of the government’s purchase of older adults services: the social undertaking capacity includes the operation of the city’s socialized market as well as the cultivation of professional talents; only when society has a strong undertaking capacity can it provide good older adults services for the older adults.

**Table 4:** Ranking of aging degree in 11 cities (districts).

Area	Data on the older adults	The degree of aging ranking
Fengtai District (Beijing)	The older adults population in the sixth district of Beijing is 2.168 million, accounting for 65% of the city’s older adults population. In Fengtai District, with the highest degree of population aging, the registered older adults population accounts for 29.2% of the total population.	The first in the municipality
Nantong City (Jiangsu Province)	Nantong is a famous hometown of longevity. The aging rate of the permanent resident population has reached 30.01%, and there are 2.318,600 permanent residents over 60 years old.	The first in the province
Wuhan City (Hubei Province)	According to the registered population information, Wuhan has the largest number of older adults, with the degree of population aging of 21.75%.	The first in the province
Anshan City (Liaoning Province)	According to the current annual increase of the older adults population in Anshan by 7.7%, it is expected that by 2020, the number of older adults aged 60 and above in the city will reach 1.03 million, accounting for about 29.4% of the total population.	The first in the province
Changning District (Shanghai)	According to the official account of Changning Civil Affairs, by the end of 2020,224,200 people registered in Changning District were registered, among which the older adults over 80 years old accounted for 6.9% of the total population of the district, ranking first in Shanghai.	The first in the municipality
Jiangning District Nanjing City (Jiangsu Province)	Nanjing has 1,824,600 permanent older adults population aged 60 or above, among which Jiangning District is the largest, reaching 216,800.	The first in the municipality
Xihu District (Hangzhou City, Zhejiang Province)	The number of older adults over 60 years old in Xihu District has exceeded 130,000, accounting for 18% of the total population.	The fourteenth in the municipality
Anqing City (Anhui Province)	The population of Anqing city aged 60 and above is 895,624, accounting for 21.50%, among which the population aged 65 and above is 710,606, accounting for 17.06%.	The third in the province
Xiashan District (Weifang City, Shandong Province)	Weihai city, which has the highest degree of aging in Shandong Province, accounts for 26.39%.	The first in the municipality
Weihai City (Shandong Province)	Weihai city, which has the highest degree of aging in Shandong Province, accounts for 26.39%.	The first in the province
Beilin District (Xi’an city, Shaanxi Province)	The population aged 60 and over was 152,839, accounting for 20.19%, of which the population aged 65 and over was 107,195, accounting for 14.16%.	The second in the municipality

## 4. Explanation of the model of factors influencing the effect of government purchase on the supply of older adults care services

Based on the theoretical analysis of the sample data, it is concluded that the factors influencing the supply effect of government purchased older adults care services are the real demand of the society formed by the high degree of aging, the complex and tense government power and responsibility system under the section logic, the government governance ability consisting of the ability to respond to problems, the ability to supply system and cooperate in governance, the digital construction ability and the supervision and management ability, and the social acceptance of older adults care services. The government’s ability to respond to problems, cooperate in the supply of institutions and governance, build digital capacity and supervise and manage, as well as the social acceptance of older adults care services, all influence the effectiveness of the local government’s purchase of older adults care services.

### 4.1. Social reality needs

In the course of the study, it was found that a general commonality among these good cases is that the degree of aging is much higher than in other regions. These regions have a stronger demand for older adults services, in which case local governments and social organizations and enterprises have a stronger external drive to provide good services for the older adults, and are therefore more likely to provide better older adults services. As shown in (Table 4), this is the ranking of the degree of aging in 11 cities (districts). Fengtai District in Beijing, with the proportion of older adults household members to the total population of the district at 29.2%, is ranked the first in the city in terms of the degree of aging; Nantong City in Jiangsu Province, a famous hometown of longevity, has an aging rate of the resident population of 30.01%, and there are 2,318,600 older adults over 60 years of age in the city, which is ranked the first in the province in terms of aging; Wuhan City in Hubei Province is ranked the first in the province in terms of its degree of aging; Wuhan City, Hubei Province, the degree of aging is also located in the province’s first … In addition to this, Anshan City, Liaoning Province, Changning District, Shanghai City, Jiangning District, Nanjing City, Jiangsu Province, Weifang City, Shandong Province, Xiaoshan District, Weihai City, Shandong Province, these five cities (districts) are also located in the province (city) first; the remaining three provinces and cities, Beilin District, Xi’an City, Shaanxi Province Of the remaining three provinces and cities, Beilin District in Xi’an City, Shaanxi Province, ranked second in the city, Anqing City, Anhui Province, ranked third in the province, and Xihu District in Hangzhou City, Zhejiang Province, ranked 14th in the province. 11 excellent cases, except for Xihu District in Hangzhou City, Zhejiang Province, the remaining 10 cities (districts) ranked in the top three of the province (municipalities) in terms of the degree of aging.

These regions are at the forefront of the aging trend in the country, with their distinctive characteristics of rapid development and large senior populations. High levels of aging mean that there is a greater likelihood of caregiving problems for the older adults, empty nesters, the disabled, the demented and even those in critical condition, which places a greater burden on family care and creates a greater demand for government purchased older adults care services. In response to the rapidly growing demand for older adults care services, the rational allocation of limited resources and the improvement of public satisfaction have become one of the pressing issues to be addressed in the government’s purchase of older adults care services (106).

Traditionally, families are the main supply of older adults care services in China, but with the miniaturization of family size, the de-feminization of women and the increased mobility of the labor force, the older adults care burden of families is getting heavier with age, and it has become difficult to support the increasing pressure on older adults care, and the family older adults care function is continuing to weaken. The large number of older adults with diverse and multi-level needs, such as living needs, medical needs, spiritual needs and mental health needs, call on local governments to introduce a series of policy measures to meet the challenges of older adults care services. Our government has long held the value of “putting people first” and some local governments, under the pressure of increasing social demand, have attached great importance to older adults care services in an effort to solve family problems and alleviate social issues. The government is actively coordinating social resources based on the resources and characteristics of the region to help improve the quality of life of the vulnerable older adults, provide support for the older adults in need, innovate the service provision mechanism and improve the effectiveness of the provision of older adults care services in order to improve the quality of life of the older adults, thus the social demand factor is the external driver for the government to purchase older adults care services.

### 4.2. The system of governmental authority and responsibility

The system of government powers and responsibilities is a fundamental proposition in the daily operation of the Chinese government, and provides a research perspective for understanding the logic of the local government’s purchase of older adults care services. Under the logic of science, the system of government powers and responsibilities is complex and tense. A sound system of government powers and responsibilities can sort out the flow of power and avoid problems such as unclear division of responsibilities and disorderly expansion of power, which is a fundamental guarantee for the effectiveness of government purchase of older adults care services. In the practice areas of these 11 excellent cases, the government’s purchase of older adults care services is generally co-ordinated by a leading group, with each (city) district and relevant departments setting up corresponding leading agencies and work teams, and the finance, establishment, audit, supervision and relevant competent departments under the unified leadership of the municipal (district) government, establishing a working mechanism under the unified leadership of the government, led by the finance department, with functional departments performing their duties and supervision departments The working mechanism of safeguarding. The improvement of the government’s power and responsibility system is conducive to each department performing its own duties, clarifying the specific division of labor, effectively performing the duties of co-ordination and planning, policy support, funding guidance, typical demonstration, supervision and management, etc., forming a practical, co-ordinated, standardized and efficient system of government purchase of older adults care services, and promoting the healthy development of government purchase of public services. For example, Fengtai District has set up a “Leading Group for Accelerating the Development of the older adults Service Industry,” headed by the executive vice mayor, with the vice mayor in charge of civil affairs, land and planning, and the office located in the District Civil Affairs Bureau, with members from relevant units; Nantong City has published the division of tasks and progress arrangements for improving the quality of older adults care services in the form of a table on its official website; Xiashan District has set up a committee headed by the District Party Committee, which is responsible for the development of older adults care services. The key tasks, leading units and cooperating units will be made public. By improving the government’s power and responsibility system and clarifying the respective work responsibilities in the leading group, the boundaries of the government’s exercise of public power and interference with private rights are outlined, and institutional adjustments and structural adjustments are made within the basic political framework and power structure, in an attempt to lock administrative power into the cage of the system. By clarifying the scope of administrative subjects and powers, regulating the basic procedures of government purchase of older adults care services, improving government supervision mechanisms and perfecting the standards of practice in the older adults care service industry, a standardized government service system is thus constructed, and the systematic promotion of holistic government construction and local government purchase of older adults care services is realised.

### 4.3. Government governance capacity

The government’s governance capacity is a direct determinant of the effectiveness of the government’s purchase of older adults care services supply. The ability to respond to problems, the ability to supply systems and cooperate in governance, the ability to build digitization and the ability to supervise and manage are together the government’s governance capacity. The specific implications are as follows: against the background of the realistic needs of society, the problem-responsiveness of local governments is the first assessment criterion of government governance capacity. Only when local governments have a strong ability to identify development opportunities, a sense of innovation and a people-oriented service concept can they have good problem-responsiveness, which is the first threshold for providing good results in older adults care services. Institutional supply capacity and cooperative governance capacity enhance the ability and willingness of multiple subjects to participate in social governance and promote the formation of a social governance community. To a certain extent, digitalization has reshaped the relationship between the government, society and market players, empowering them with a “multi-faceted and collaborative” governance mechanism. Supervisory and management capacity is the last line of defense to fully utilize the power of social participation to enhance the effectiveness of social governance and limit its negative effects. The government, society and families are the three key stakeholders involved in the government purchase of older adults care services, and it is clear that the key party in resolving conflicts is still the government. The government plays an important role in the whole process as the responder to the issue of government purchase of services, the initiator of service supply, the provider of funds, the coordinator of cooperative governance and the ultimate regulator. Although the government’s measures to purchase community-based older adults care services have alleviated the pressure to respond to the demand for older adults care services, the ‘public nature’ of older adults care services prevents the government’s responsibility from being weakened (71). The government should take “unlimited responsibility” for the sound functioning of the mechanism, including institutional improvement, financial incentives, coordination of interests, and monitoring and evaluation (107). The socialization of intergenerational relationships is also giving rise to new models of market provision and family support for older adults care services (108). In the operational mechanisms of the 11 outstanding cases, the degree of market and social participation, as well as the form and state of participation, depend mainly on the operational space given by the government, and the local government triggers the strategic choices of society and families to evolve in the direction of ‘provision’ and ‘participation’, continuing to play a role in the mechanism The local government’s strategic choice of triggering society and families evolves in the direction of ‘supply’ and ‘participation’, and continues to play a leading role in the design of mechanisms and the construction of policies to promote win-win benefits for all participating parties.

### 4.4. Social acceptance capacity

The ability to take over is the basis for social organizations to effectively participate in the supply of older adults care services, and the ability to take over determines the direct supply effect of the local government’s purchase of older adults care services. At the present stage, a common problem facing China is that the lack of social organizations has seriously restricted the vigorous implementation of the government’s purchase of public services (109), and the “inability” and “necessity” are the real dilemma faced by social organizations in the government’s purchase of older adults care services. This is the real dilemma faced by social organizations in the government’s purchase of older adults care services. In the 11 cases studied in this paper, local governments were able to mobilize their own social resources to provide support for older adults care services, and the role of incentives and guidance was obvious. Social organizations are more capable in terms of funding, personnel, rules, technology, knowledge and skills, and are more familiar with the characteristics and real needs of the public served, which supports the good supply effect of government-purchased older adults care services. Social forces have shown considerable autonomy and feasibility in the process of government purchase of older adults care services, caught in the complexity of the relationship between interest subjects, forming a government-led partnership with the government to provide a richer range of older adults care services in a public demand-oriented society. Under the market logic, social organizations and enterprises are often faced with risks and challenges in multiple dimensions, such as organization, operation, system and ethics. They are able to build up a good relationship of trust with the public under the supervision and control of the government, to develop their innovative capacity, to improve their operational efficiency and to optimize the effectiveness of the provision of older adults care services.

## 5. Conclusion and discussion

The government purchase of older adults care services is a large and complex system project, designing many elements and systems. This paper focuses its attention on the influencing factors of the service supply effect, and studies 11 excellent cases of local government purchase of older adults care services, including Fengtai District in Beijing, coding the collected data at three levels with the help of the rooting theory approach to the content of the data, and constructing a relational model. Text analysis was conducted with the help of NVivo12 software in an attempt to answer the question: Why did the local governments’ purchase of older adults care services show differentiated effects when they were also driven by high central policies, constrained by limited government resources and with the same weak economic preference attributes? What are the factors that influence the supply effect of local government purchase of older adults care services? 11 cases, although the service models show diversified, differentiated and diverse characteristics, but through the extraction of the primary data, it can be concluded that the regions with good supply effect of government purchase of older adults care services have the same generation logic and supply mechanism. Based on the logic of the local practice of local government purchase of services, this paper abstracts the “social reality demand,” “government power and responsibility system” and “government governance capacity” around the story line of “the effectiveness of local government purchase of older adults care services supply.” “Government governance capacity” and “social acceptance capacity,” which have significant effects on the supply effect of government purchase of older adults care services, and further proposes that the factors influencing the supply effect of local government purchase of older adults care services and causal mechanisms are as follows:

The study found that the effect of local government purchasing on the supply of older adults care services is clearly driven by the social reality of demand triggered by the degree of aging, and the higher the degree of aging, the stronger the external drive of the government, and the more likely it is that the local government will be motivated to provide better older adults care services. At the same time, the purchase of older adults care services by local governments needs to focus not only on the external variable of real demand, but also on the internal variables of local governments and the solidity of the legitimacy base. The complete system of governmental authority and responsibility under the section logic, with a clear division of responsibilities, provides a fundamental guarantee for the effectiveness of the service, ensuring that every aspect of the government’s purchase of older adults care services runs on the right track. The outsourcing of older adults care services has changed the role of the government from that of an ‘athlete’ to that of a ‘referee’, putting the government’s governance capacity to a higher test. The ability to respond to problems, supply systems, cooperate in governance, build digitalization and supervise and manage is an important manifestation of the government’s ability to govern in five areas. Social organizations and enterprises play an essential role in supporting the government’s purchase of older adults care services. The government uses flexible governance tools to tap into informal resources and guide and encourage social forces to participate in the older adults care services industry, thereby improving the operational efficiency of the older adults care services industry and enhancing the effectiveness of service provision. It can be seen that in the government purchase of older adults care services, the government and society show a strong dependency relationship, and the practical tension between the double logic of the section and the market gives rise to a win-win taking direction for the organization’s operation, thus constituting a practical path for the government purchase of older adults care services supply, and the “section-market” the legitimacy and efficiency of the dual interaction mechanism is the key to the success of the local government’s purchase of older adults care services.

As an important means for the Government to guide and supervise social forces, policy tools play a key role in promoting the development of the older adults services industry, and policy orientation and decision-making will have a significant impact on the quality of the supply of older adults services for the older adults and the development of the older adults services industry. Promoting the synergistic development of the government and social forces through policy will help mature and finalize the system of social services for the older adults and help improve the effectiveness of older adults services. The government needs not only to mobilize social forces and rationally integrate resources, but also to guide and supervise the process of purchasing older adults services through policy instruments. Therefore, the government should make good use of welfare policy tools, formulate a series of older adults service policies, improve the government’s system of purchasing older adults services, and clarify the direction of policy guidance, so as to give full play to the professional advantages of social forces’ services, and at the same time reflect the integrative efficacy of the government’s governance. In short, the sense of gain, happiness and security of the older adults group is the starting point and landing point of the older adults service policy formulation and implementation. The development of older adults services is related to social stability and sustainable economic development, as well as the well-being of people’s lives. Optimizing the overall layout of long-term care policies for the older adults and promoting the development of the government’s policy system for purchasing older adults services are of great significance to the implementation of the national strategy for actively coping with population aging.

Current academic research on the government’s purchase of older adults care services focuses on the synergistic relationship between the government and society, the government’s decision-making and behavior in the process of purchasing older adults care services, and the imbalance between the government’s supply and the demand for individual care services for the older adults care, which is enlightening for a deep understanding of the operation mechanism of the government’s purchase of older adults care services. The shortcomings lie in the fact that the current research is generally characterized by fragmentation, lack of description of diverse practices in different regions, insufficiently comprehensive and in-depth research on the whole process of government purchasing of older adults care services, failure to put forward systematic theoretical models, and lack of refinement of the universal logic of beneficial experiences. Therefore, on the basis of previous research, this paper further asks, what are the influencing factors of the effect of local government purchasing older adults care service supply? Why are there differentiated effects of older adults care services in different regions? What are the causal mechanisms in those effective local governments purchasing older adults care services? This paper adopts the grounded theory research method, starting from the practical experience of Chinese regions, selects 11 typical representative cases of government purchase of older adults care services from the list of excellent cases of reforming home and community-based older adults care services announced by the Ministry of Civil Affairs, establishes theories on the basis of empirical data, summarizes the experiences in the original data and then rises to theories, and responds to the factors influencing the effect of local governments’ purchase of senior care services on the doctrinal level. The study responds to the factors affecting the effectiveness of local governments’ purchasing of older adults care services at the theoretical level as well as the internal causal mechanism, and explores the paths for local governments to improve the effectiveness of their purchasing of older adults care services under the Chinese institutional environment, which enriches the relevant knowledge of older adults care services, and provides a realistic reference and a kind of theoretical interpretation for the “Chinese purchasing of older adults care services” during the period of transition. It also provides a reference for the practice of government purchasing of older adults care services in other countries around the world.

## Data availability statement

The original contributions presented in the study are included in the article/supplementary material, further inquiries can be directed to the corresponding author.

## Author contributions

SL: this study was not funded by any grants.

## Conflict of interest

The author declares that the research was conducted in the absence of any commercial or financial relationships that could be construed as a potential conflict of interest.

## Publisher’s note

All claims expressed in this article are solely those of the authors and do not necessarily represent those of their affiliated organizations, or those of the publisher, the editors and the reviewers. Any product that may be evaluated in this article, or claim that may be made by its manufacturer, is not guaranteed or endorsed by the publisher.

## Footnotes

^1^http://www.gov.cn
